# Controlling liver cancer internationally: A qualitative study of clinicians' perceptions of current public policy needs

**DOI:** 10.1186/1478-4505-9-32

**Published:** 2011-07-28

**Authors:** John FP Bridges, Gisselle Gallego, Barri M Blauvelt

**Affiliations:** 1 Department of Health Policy and Management, Johns Hopkins Bloomberg School of Public Health, Baltimore, Maryland, USA; 2 Institute for Global Health, University of Massachusetts, Amherst, Massachusetts, USA

## Abstract

**Background:**

Liver cancer is the fifth most common cancer in men and the seventh for women. Usually because of late diagnosis, the prognosis for liver cancer remains poor, resulting in liver cancer being the third most common cause of death from cancer. While some countries have treatment guidelines, little is known or understood about the strategies needed for liver cancer control internationally.

**Objective:**

To explore leading liver cancer clinician's perceptions of the current public policy needs to control liver cancer internationally.

**Methods:**

Key informant interviews were conducted with a range of liver cancer clinicians involved in policy in eleven countries. Interviews were digitally recorded, transcribed verbatim, translated (where necessary), de-identified and analyzed by two researchers using a constant comparative method.

**Results:**

Twenty in-depth semi-structured interviews were conducted in: Australia, China, France, Germany, Italy, Japan, Spain, South Korea, Taiwan, Turkey and the United States. Nine themes were identified and cluster into three groups: 1) Promoting prevention via early risk assessment, focusing on viral hepatitis and other lifestyle factors; 2) Increasing political, public and medical community awareness; and 3) Improving funding for screening, liver cancer surveillance and treatment.

**Conclusion:**

This study is an important step towards developing an evidence-based approach to assessing preparedness for implementing comprehensive liver cancer control strategies. Evaluation mechanisms to assess countries' performance on the needs described are needed. Future research will concentrate of understanding how these needs vary across countries and the optimal strategies to improve the diagnosis and prognosis of patients with liver cancer internationally.

## Introduction

Liver cancer is the fifth most common cancer in men and the seventh in women, because of its high fatality it is the third most common cause of death from cancer worldwide [1]. Hepatocellular carcinoma (HCC) is the predominant histologic subtype compromising approximately 85-90% of all primary liver cancers [2]. In 2008 there were an estimated 695 000 deaths from HCC globally among whom at least two thirds of these were in the Asia Pacific region [3]. Most HCCs are discovered late in advance stages due to the relative dearth of symptoms in early stages and the rapid doubling of the tumor [4]. Median survival of patients is estimated at less than a year and less than five months without effective treatment [5].

Clinical guidelines have been developed by the American Association for the Study of Liver disease (AASLD), the European Association for the study of the liver (EASL), the Asian Pacific Association for the study of the liver (APASL) and the Japanese society of hepatology. According to these guidelines potentially curative treatment for patients with early stage HCC include surgical resection, percutaneous ablation and liver transplantation. Fewer effective options are available for those with an advanced disease [6-9]. However as noted by Lin and Kao, the guidelines differ from one another and further research is needed to develop best practice guidelines for optimal management of HCC [10].

While clinical guidelines are important and sometimes effective in standardizing care, [11] a broader scope is needed to address what is described as a public health problem in need of cohesive strategies to ensure its proper prevention, control and management [12]. Unlike clinical guidelines, national policies for comprehensive liver cancer control, from prevention, early detection, and treatment are not widely available. Moreover, little is known or understood about the current public policy needs for cancer prevention and control in many countries around the globe. Furthermore a consensus statement from the Asian Oncology Summit highlights the need for "policies directed at reducing risk factors for HCC" [13].

In recent years policy makers have acknowledged liver cancer as a significant public health problem [3,13,14]. The real impact however, is clouded by the lack of reliable epidemiologic surveillance data. For example a report from the European Hepatitis B expert group noted the there is a scarcity of reliable epidemiological data on hepatitis B, a leading cause of liver cancer [15]. Despite the calls to focus on these difficulties, the complexities of liver cancer and the limited political and public awareness make it complicated to identify the most significant needs to address this growing problem. This paper aims to identify the needs impacting liver cancer research, prevention and treatment, especially focused on hepatocellular carcinoma (HCC), across Asia, Europe and North America. The study qualitatively explored leading liver cancer clinician's perceptions of the current public policy needs to control liver cancer.

## Methods

### Respondents and recruitment

Key informant interviews were conducted with a range of liver cancer clinicians purposively sampled on the basis of being involved in policy and in liver cancer and related disease prevention, detection, and management. They were identified using the following criteria: i) actively working in liver cancer in their country; ii) involved in HCC clinical practice and policy or research; iii) active members in national and international liver associations and/or published extensively in peer review journals. Potential respondents were informed about the study via email or mail, and received a follow up telephone call or email if they did not respond within two weeks. The email or mail invitation was sent in English as well as in the national language of the country, where necessary. Upon a positive response, the interviewer requested basic demographic information and scheduled an appointment.

### Data collection and analysis

An interview guide was used as a prompt sheet ensured the same topics were covered during the interviews. However questions were not asked in a standard way and respondents were able to articulate their own perceptions of current liver cancer policies and strategies to advance liver cancer prevention, treatment and research. Respondents were asked to comment on their countries' current needs in combating liver cancer and major policy issues impacting diagnosis and prognosis.

Invitations to participate were sent to twenty five people. The invitation outlined the objectives of the study, explained the characteristics of the interview process (open and flexible), informed participants about the potential benefits and harms, ask for consent to record the interview and informed about the founder of the study and the lack of remuneration for participation.

In-depth, semi-structured interviews were carried out between February and June 2010. The interviews were conducted with individual respondents either in person or by telephone in English or in the respondent's native language by trained fieldworkers with extensive experience conducting medical/scientific interviews in the relevant countries. When conducted in the interviewee's native language, the field notes were immediately translated into English by the interviewer fluent in both languages. Interviews were digitally recorded, transcribed verbatim, translated (where necessary), de-identified and analyzed. One person refused and four other accepted but were not interviewed as thematic saturation was reached after the twentieth interview.

Preliminary data analysis was conducted after each interview. This allowed identification of issues that required further exploration in the interviews that followed. Continuous analysis of collected data was performed. After preliminary analysis was performed, segments (paragraph, sentences) were coded and labeled (i.e. data extracts from each transcript were grouped together under the appropriate category). Coded segments were then compared for differences and similarities of events and ideas. This process was repeated until all comments were assigned to categories (constant comparison) [16]. Data were validated in two different ways. More than half of the interviews were coded on two separate occasions to ensure consistency with the coding. Categories were reappraised by the researcher to judge whether any data had been misplaced (negative case testing) [17].

### Ethics

All participants were informed about the study and its potential risks and benefits. Participation in the study was voluntary and respondents were not reimbursed for participation. The study was deemed exempt from human subject's consideration from the Johns Hopkins University, Bloomberg School of Public Health Institutional Review Board (IRB). Local experts were also consulted to verify if any additional country specific requirement needed to be met and the study also complied with all local regulations.

## Results

**Figure 1:**
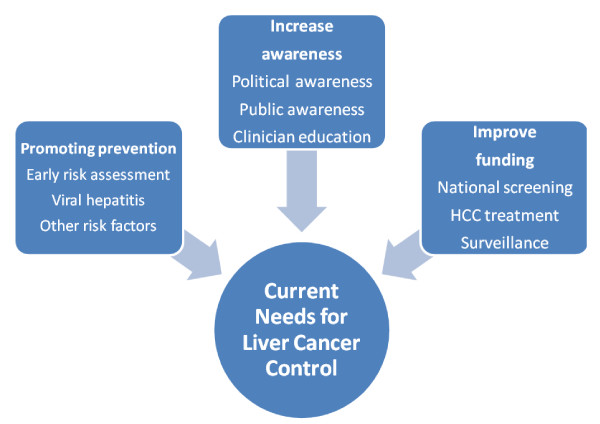
**Current needs for liver cancer control**.

Twenty in-depth semi-structured interviews were conducted with leading liver cancer clinicians in 11 different countries: Australia (n = 1), China (n = 1), France (n = 1), Germany (n = 3), Italy (n = 1), Japan (n = 2), Spain (n = 1), South Korea (n = 2), Taiwan (n = 1), Turkey (n = 3) and the United States (n = 4). Interviews lasted between 16 and 80 minutes (mean time 34 minutes) and elicited a broad range of perceptions about public policy needs to control liver cancer internationally. As shown in Figure 1 three major themes emerged: the need to promote prevention, increase awareness and improve funding. Table 1 summarizes these themes and the dimensions contained in each one. Each of the themes and dimensions are further discussed in the following text.

**Table 1:** Themes and dimensions

Theme	Dimension	
Promoting prevention	Viral hepatitis	*"You really need to work backwards in the spectrum of the disease so you are detecting more early disease and either curing Hep C or preventing Hep B both of which will eventually lead to a decreased incidence of HCC"*.
	
	Early risk assessment	*"We need to go out and identify at-risk patients, encourage them to be screened, and linked them to appropriate care"*.
	
	Other risk factors	*"Part of the problem is that the prevalence of obesity continues to rise, it may have leveled off now, but until now it has been rising precipitously so that there will presumably be a lag period during which patients who are obese and then over time they will accrue a risk of liver or fibrosis and cancer"*.

Increase awareness	Political awareness	*"...on a national basis the government has no strict plan for HCC and this is a big issue"*.
	
	Clinical education	*"Primary care doctors need to be sensitized to the risk of liver disease, the implications, the detection in particular of viral hepatitis, the treatment options, and the need to refer, and ultimately screening for liver cancer"*
	
	Public awareness	*"There is a total absence of public awareness as best as I can tell. I am not there with my finger on the pulse of the public, but it's fairly clear that this never gets on the radar the way breast, colon, melanoma, pancreas, and other common cancers do"*.

Improve funding	Screening	*"There's a clear need for screening of cirrhotic patients. Screening is expensive......"*.
	
	Treatments	*"...the new and expensive drugs and technologies are an exception. So many patients complain, many patients ask for a new technology or a newly developed drug and treatment. However they are not covered by insurance, so that's a big problem"*.
	
	Surveillance	*"a more concerted effort to coordinate with [Ministry of Health] and to develop more robust prevalence data about viral hepatitis worldwide"*.

### Theme 1: Promote prevention

All participants described promoting prevention as an important need in liver cancer control. There were three dimensions of this theme: The need to prevent viral hepatitis (primary prevention), early risk assessment for HCC, and management of other risk factors. This is summarized in one of the respondent's quotes:

*"Needless to say, prevention will reduce the number of cases. Prevention of infection of hepatitis B and C comes first. Next, prevention of cancer, screening, complete cure treatment, prevention of reoccurrence, and early diagnosis and treatment of reoccurrence: This will eliminate cancer. So, prevention comes first"*.

#### Viral hepatitis

Prevention of hepatitis B and C virus (HBV and HCV) infection was described by all respondents as an important step towards reducing the incidence of HCC. Many indicated that most countries in the study have a well-established, nationwide vaccination program for HBV with some countries already demonstrating reductions of HCC and respondents expecting the incidence of HCC to further decrease in the next 10-20 years. This perception is supported by a 20 year follow up study of hepatitis B vaccination in Taiwan [18]. However, respondents also described that despite this, a vast number of people will still develop acute and chronic hepatitis B. In addition, even in countries with national vaccination policies, respondents commented that there still are portions of the population that may not be vaccinated or protected.

It is interesting to note that in one of the countries, the uptake of hepatitis B vaccination is quite low (described as 20-30%). This is due to the public's perception that vaccines are linked to neurological disorders. The respondents in this country also commented that these perceptions were fuelled by the media.

*"The media and the newspapers several years ago popularized the notion that the vaccine is responsible for neurologic disorders. Therefore the vaccination against HBV has been widely unpopular and has been stopped at times"*.

Participants described some high risk populations as foreign born (especially from those countries where hepatitis B vaccination was not available to them at the time of their birth), and past or current injection drug users. Therefore, control of both HBV and HCV is still an issue in most countries. This is consistent with a recent report by the Institute of Medicine (IOM) in the United States which states that *"unless action is taken to prevent chronic hepatitis B and hepatitis C, thousands more Americans will die each year from liver cancer or liver disease related to these preventable diseases" *[19].

#### Early risk assessment for HCC

The majority of respondents described "early risk assessment" as paramount in liver cancer control. This was considered by most as particularly important, considering that early detection and treatment of liver disease have a major impact on HCC survival outcomes.

"We can actually identify the high risk patients and use the well- developed screening strategies to monitor these patients, so that these patients, if they ever develop hepatocellular carcinoma, can actually be diagnosed at the early stage, and treated..."

To enable the health care system to successfully intercept and control liver disease at early stages, some respondents call for primary care doctors to screen and treat viral hepatitis and also to screen for and manage risk factors associated with liver cancer. *"Primary care doctors need to be sensitized to the risk of liver disease, the implications, the detection in particular of viral hepatitis, the treatment options, or the need to refer, and ultimately screening for liver cancer"*.

Most respondents described that liver cancer is rarely detected at an early stage, limiting the amount and effectiveness of treatment and other efforts to better manage it. As noted by one participant *"half of the cases of HCC are diagnosed outside of screening programs, which is an incredibly low figure - meaning that patients are not detected in early stages"*.

#### Other risk factors

A number of respondents described that besides heavy alcohol consumption, obesity and diabetes are risk factors that could be modified and have an impact on HCC incidence. Non-alcoholic steohepatitis (NASH) was also mentioned as an important risk factor. Some respondents noted that thanks to vaccination and better needle exchange practices, alcohol-induced hepatitis, obesity, diabetes mellitus and metabolic syndrome will be the main causes of liver cancer in the future. However various respondents mentioned that more research is needed to understand this relationship as these conditions often occur in tandem. Consistent with the respondents comments, Stickel et al, have described the increased incidence of HCC in patients with non-alcoholic fatty liver disease (NAFLD) which is associated with metabolic syndrome, obesity and insulin resistance [20].

*"I would say the first thing is really to improve the awareness with regard to potential risk factors. These include the obvious, hepatitis infection with B and C, lifestyle modification to reduce alcohol, and make sure that we know the epidemiology trend for non-alcoholic fatty liver disease and the potential impact on HCC incidence"*.

### Theme 2: Increase Awareness

The second theme that emerged from interviews with the key informants was centered on increasing awareness. Participants commented that liver disease and HCC knowledge and awareness was poor amongst three different groups: politicians, the general public and doctors.

*"The low level of provider knowledge around viral hepatitis...that is a cascade of problems, people not getting vaccinated, people not getting information about the value of screening, people not getting screened, misinterpretations of diagnostic or screening tests, misinformation like hepatitis B is not going to kill you, it's in an inactive state, etc. and then referrals not being made to treatment centers to get treatment"*.

#### Political awareness

Most respondents considered that chronic hepatitis B and C need to be recognized as an important public health problem, but that due to other, more acute health priorities and well-coordinated public and political demand, it is hard to gain support of policy-makers and political bodies involved in supporting funding and prioritizing liver cancer.

"The principal gap for HCC is the absence of a common policy in all regions [of the country]."

*"Liver cancer is not regarded as a major health problem. It is regarded as a rare cancer. There are no public policy initiatives"*.

*"The lack of a national approach" *was quoted by some of the respondents. This was sometimes linked to the lack of accurate data on incidence and prevalence, but more often mentioned as related to the lack of a well-coordinated and linked effort by different interests and initiatives related to liver disease.

#### Public awareness

The majority of respondents commented that visibility for liver cancer is low compared to other major cancers such as colon, breast, and lung. The public's understanding of liver cancer and knowledge about the risk factors were described as limited. "*There's absolute ignorance among [the] common population and there is a clear need for education"*.

According to most respondents, public awareness must be increased *"to let people know that chronic liver disease is a major risk factor for HCC"*. Public health campaigns are needed to stress the importance of knowing risk factors and to encourage early detection of liver disease and cancer. Several respondents noted the importance of health campaigns and media exposure to increase awareness amongst the general public about chronic viral hepatitis. Some respondents attributed the lack of public awareness to the absence of advocacy.

*"There are more gaps than advocacy at this point, meaning that there is certainly no coordinated public awareness; there's barely awareness of hepatitis screening, detection, and treatment"*.

#### Clinical education and awareness

Some of the respondents mentioned that the medical community at large should be educated. Several respondents specifically referred to the need to educate doctors in primary care settings to proactively asses and manage liver disease risk factors, and also to refer such patients to centers of excellence in hepatology and liver cancer.

*"Primary care doctors need to be sensitized to the risk of liver disease, the implications, the detection in particular of viral hepatitis, the treatment options, or the need to refer, and ultimately screening for liver cancer"*.

### Theme 3: Improve funding

The third theme was *funding *including dimensions related to funding and reimbursement of screening, surveillance and treatments.

#### Screening

As previously stated, some respondents described the need for nationwide screening programs for HCC. A number of respondents mentioned screening both for viral hepatitis and also *"screening for cirrhosis *[Non-HBV] *in the general population"*. Several respondents considered that resources need to be better allocated for such programs. Increased political (government) awareness and the development of mandatory screening guidelines and systems would improve this process.

*"Definitely you need to have some policy changes at the government level... funding is a major consideration"*.

Bruix and Llovet described how HCC meets most of the criteria that justify a screening program: "the population at risk is known, the disease is highly prevalent, it has a high mortality, and effective screening tests are available and acceptable" [21]. However as stated by some respondents and identified in the literature, there are issues around sensitivity and specificity of screening tests (i.e. Alpha-Fetoprotein (AFP)), the screening interval and the impact of screening on HCC survival [22]. Furthermore Gomma al colleagues have identified that although there is an international consensus on the diagnostic pathway for HCC, there is no "ideal screening modality" [23].

#### Surveillance

Most respondents also identified the need for surveillance to document the incidence and prevalence as well as the burden of liver disease and cancer. Current efforts to collect data seemed to be supported by "champions" and on a voluntary basis. "*We have already started a nationwide liver cancer registry, for more than five years now. It is voluntary data. More than 20 institutes are taking part. But because of a limited budget, actually, it is not so active." *However it is important to note that some countries do collect data in a systematic and comprehensive way. Consistent with the views given by the respondents about the importance of surveillance, a study on HCC screening conducted by Zhang et al, found that surveillance of hepatitis B carriers in shanghai, China reduced HCC related mortality by 37% [22].

#### Treatments

Some respondents reported the lack of insurance coverage for "targeted therapy". A number of respondents noted that new therapies are likely to have a high cost, thus limiting patient access.

*"The advent of many new drugs will hike up the medical cost and, since these drugs are produced by foreign pharmaceutical companies, the money [name of the Country] will flow out of the country. Therefore, I think this will have a serious effect on the policy of medical costs"*.

Most respondents described the importance of collecting the *"appropriate data" *which could inform policy makers about the social burden of the disease and allow them to allocate resources into prevention and control of liver disease and cancer.

"We don't yet have a generally accepted surveillance strategy to detect and uncover patients with increased risks, nor have an organized surveillance program in which these individuals could enter to get regular check-ups of the liver."

## Further discussion

Even though there are differences in terms of resources, the etiology of the disease and access to health care, the needs for liver cancer control and implications for policy described by the respondents in this study were similar. In particular, some respondents consider there is still a need for a global (international approach) to liver cancer control. *"An authority [such as] the World Health Organization, can institute some coordinated (approach) with the government policies within individual countries." *Even this may not be enough. Some of the respondents recommended having a global approach that raises awareness about the disease.

*"I think you need to let the message out, because I think people need to know how significant this disease is, both here and globally. I think we need to have some comparison about the outcome and how many lives we can save if we improve the public awareness for liver versus some other more common cancers. Hopefully...we can do this kind of comparison so that people all of the sudden realize this disease may not be as trivial as people thought"*.

It is important to note some limitations in the study. First, it was conducted among leaders from a limited set of countries and, consequently, the findings may differ in other country settings. Second, reporting of needs in liver cancer care, and policy may have been influenced by respondents' greater knowledge of one area as opposed to other areas. Thirdly, a greater diversity of respondents may identify additional needs and suggest further strategies for a more systematic approach to liver cancer control.

## Conclusion

According to the stakeholders in this study, different needs (improve prevention, awareness, funding) were identified to formulate a comprehensive liver cancer control strategy. Even though health policy varies from country to country and different countries have specific needs and views, this study showed that there were more common than dissimilar needs in the countries that participated. The "needs" identified by the respondents refer to the gaps in prevention, treatment of liver disease and other strategies necessary to achieve liver cancer control.

To our knowledge this is the first study explore leading liver cancer clinician's perceptions of the current public policy needs to control liver cancer internationally. This exploratory study is an important step towards developing an evidence-based approach to assessing preparedness for implementing comprehensive liver cancer control strategies. Evaluation mechanisms to assess countries' performance on the needs described are needed. Future research will concentrate of understanding how these needs vary across countries and the optimal strategies to improve the diagnosis and prognosis of patients with liver cancer internationally via a. a quantitative survey.

## Competing interests

The authors declare that they have no competing interests.

## Authors' contributions

JB led the study, its design, analysis of the data and drafting of the manuscript. GG conducted the data analysis and drafting of the manuscript. BB participated in the development of the study, data collection, the interpretation of the data and editing of the manuscript. All authors read and approved the final manuscript.

## Funding

This study was supported by funding from Bristol Myer Squibb (BMS). The funders had no role in study design, data collection and analysis, selection of respondents, decision to publish, or preparation of the manuscript.

## Appendix 1. Qualitative interview protocol

### Needs assessment

1. First, can you tell me a little about your nation's strategies to advance liver cancer prevention, treatment and research?

2. What do you see as the main gaps in public policy in your nation?

#### Aide memoire

a. Gaps at the national or federal

b. Interaction at the international level

c. Supporting patient advocacy efforts

d. Public educations and preventions strategies

e. Funding and organization

3. I would now like to focus on your nation's current needs for infrastructure associated with liver cancer prevention, treatment and research.

#### Aide memoire

a. Research facilities

b. Primary services

c. Secondary services

d. Tertiary services

e. Prevention

4. Let us now focus on your nation's current educational needs associated with liver cancer prevention, treatment and research.

#### Aide memoire

a. Physician training

b. Nurse training

c. Researcher training

d. Patient education

e. Population training

5. Can we talk now about any evidence gaps that exist in your nation relating to liver cancer?

#### Aide memoire

a. Prevalence/surveillance

b. Burden of disease

c. Effective and cost effective prevention strategies

d. Effective and cost effective treatment strategies

e. National or international guidelines
